# Are You Willing to Self-Disclose for Science? Effects of Privacy Awareness and Trust in Privacy on Self-Disclosure of Personal and Health Data in Online Scientific Studies—An Experimental Study

**DOI:** 10.3389/fdata.2021.763196

**Published:** 2021-12-24

**Authors:** Cornelia Herbert, Verena Marschin, Benjamin Erb, Dominik Meißner, Maria Aufheimer, Christoph Bösch

**Affiliations:** ^1^ Department of Applied Emotion and Motivation Psychology, Institute of Psychology and Education, Ulm University, Ulm, Germany; ^2^ Institute of Distributed Systems, Ulm University, Ulm, Germany

**Keywords:** self-disclosure, privacy paradox, human-centered privacy, mental health, university students, trust in privacy, privacy awareness, human factors

## Abstract

Digital interactions via the internet have become the norm rather than the exception in our global society. Concerns have been raised about human-centered privacy and the often unreflected self-disclosure behavior of internet users. This study on human-centered privacy follows two major aims: first, investigate the willingness of university students (as digital natives) to disclose private data and information about their person, social and academic life, their mental health as well as their health behavior habits, when taking part as a volunteer in a scientific online survey. Second, examine to what extent the participants’ self-disclosure behavior can be modulated by experimental induction of privacy awareness (PA) or trust in privacy (TIP) or a combination of both (PA and TIP). In addition, the role of human factors such as personality traits, gender or mental health (e.g., self-reported depressive symptoms) on self-disclosure behavior was explored. Participants were randomly assigned to four experimental groups. In group A (*n* = 50, 7 males), privacy awareness (PA) was induced implicitly by the inclusion of privacy concern items. In group B (*n* = 43, 6 males), trust in privacy (TIP) was experimentally induced by buzzwords and by visual TIP primes promising safe data storage. Group C (*n* = 79, 12 males) received both, PA and TIP induction, while group D (*n* = 55, 9 males) served as control group. Participants had the choice to answer the survey items by agreeing to one of a number of possible answers including the options to refrain from self-disclosure by choosing the response options “don’t know” or “no answer.” Self-disclosure among participants was high irrespective of experimental group and irrespective of psychological domains of the information provided. The results of this study suggest that willingness of volunteers to self-disclose private data in a scientific online study cannot simply be overruled or changed by any of the chosen experimental privacy manipulations. The present results extend the previous literature on human-centered privacy and despite limitations can give important insights into self-disclosure behavior of young people and the privacy paradox.

## 1 Introduction

Scientist, companies and institutional organizations are able to collect, monitor and analyze vast amounts of digital data via the internet from users all over the globe. Web-based computer-assisted communication and digital interaction between humans or between humans and machines or between humans and virtual agents have become the norm rather than the exception in our digitalized global society. With personal computers, smartphones, tablets, and other computing devices, communication has become ubiquitous—thus enabling data tracking, data collection, and data exchange in arbitrary situations at home or at work. As part of this digitization progress and world-wide use of internet-based communication and data processing, the internet user’s self-disclosure behavior as well as the user’s understanding, awareness of and trust in data privacy have become prominent topics and fields of scientific research (Barhamgi et al., 2021). Summarized under the umbrella terms of *user-centered* or *human-centered privacy* these topics have received broad interest in ethics, law, computer science, psychology, life, and social sciences alike.

Recent surveys and investigations of self-disclosure behavior on internet platforms are in fact alarming: as observed in a recent survey by e.g., Bitkom (one of Germany’s digital associations), only 3% of the survey-volunteers reported to care about the privacy of their internet data. The majority of volunteers (87%) reported to use online services although they do not have full confidence that the services comply with the legal requirements and standards of data protection. Almost 31% replied to not even care about the compliance of the service with data protection (Bitkom, 2015). The findings mirror a striking paradox: albeit internet users worry about their data, they display a high willingness to openly self-disclose by sharing private data on the internet. This paradox (worry about privacy despite personal data disclosure on the internet) has been investigated under the label *privacy paradox* (Bedrick et al., 1998; Barnes, 2006). The privacy paradox describes the discrepancy between the users’ self-reported attitudes, i.e., their concerns, worries, and fear about privacy violations on the internet and their motivation to protect themselves against it, for example by investing time and effort in self-regulated control of data protection security. The privacy paradox has found evidence in many studies so far, although not without doubt (e.g., Acquisti and Grossklags, 2005; Dienlin and Trepte, 2015). While appropriate technical data protection mechanisms prevent illegitimate access to collected data by third parties, the service providers themselves often have a strong motivation to collect as much data as possible for their own purposes (Bösch et al., 2016). Ethically, individuals have the fundamental right of protection of their privacy. Therefore, privacy-friendly providers (both commercial and scientific) must take adequate steps to protect the privacy of their users including the confidentiality of the user’s data. From a psychological perspective, the development of privacy-preserving computer applications and services needs to take into account the human factors related to privacy and privacy decision making. Specifically, knowing which human factors most significantly influence self-disclosure behavior on the internet is a prerequisite for establishing user-aware and trustworthy data sharing via the world-wide web (www). In fact, thoughtless handling of private information can have negative effects for both, the user and the service provider (Boyd and Ellison, 2007).

Psychological research provides first answers to the questions about self-disclosure on the internet (for an overview, e.g., Joinson and Pane, 2007). Many studies suggest that self-disclosure behavior increases in computer-mediated communication compared to face-to-face communication. The results suggest that anonymity and the absence of a social human presence both facilitate self-disclosure. This relationship between anonymity and absence of human presence holds true for self-disclosure in online surveys, for self-disclosure in web forums and extends to people’s willingness of increased self-disclosure while talking to virtual agents compared to talking to real healthcare professionals (for an overview, e.g., Joinson and Pane, 2007). Therefore, the users’ motivation to self-disclose, their expectations of anonymity and their perception of social contextual factors including the *Why*, *Where* and *with Whom* to self-disclose are all relevant human factors that human-centered privacy approaches should take into consideration. This is necessary, because these human factors seem to facilitate or reduce the privacy paradox mentioned above: for example, in a recent meta-analytic study (Baruh et al., 2017), it has been found that the users’ worry about privacy on the internet and the degree of personal data they share on social media is inversely related. This was found across several studies (Baruh et al., 2017). According to psychological theories of planned behavior, the self-disclosure behavior of users can be explained by their motivation and expectancies about the rewarding aspects of data sharing (outcome belief), their perception of the self-disclosure behavior of others being in the same situation (normative belief), and the degree of self-control (control belief) (Dienlin and Trepte, 2015; Trepte et al., 2020).

Privacy awareness (PA), trust in privacy (TIP), as well as personality traits (cognitive, affective, and motivational) might influence the user’s willingness of sharing personal data on the internet. Privacy awareness (PA) comprises different facets related to the user’s attention, perception and cognitions of privacy (for an overview, e.g., Pötzsch, 2009). Trust in privacy (TIP) relates to the user’s confidence that the data provided or shared will not be exploited for unknown purposes during or after the process or after the transmission of the data (see Wirtz and Lwin, 2009). PA and TIP both can influence the user’s intentions of self-disclosure, either implicitly or explicitly, depending on how they are presented on the internet or manipulated experimentally: on the one hand, PA and TIP can be presented in a situation- and user-dependent manner, for example by providing direct information and feedback to the user on the website. On the other hand, PA and TIP can be induced in a situation- and user-independent manner, for example by launching general campaigns to support privacy literacy in general (Pötzsch, 2009; Wirtz and Lwin, 2009). Theoretically, PA and TIP might influence self-disclosure positively or negatively and independently from each other or in combination with each other (Pötzsch, 2009; Wirtz and Lwin, 2009; Taddei and Contena, 2013).

Therefore, knowing how PA and TIP influence self-disclosure behavior on the internet becomes a critical instrument for many internet services that allow the user to share sensitive information about their mental or physical health, for example, in an internet forum or on a scientific research platform. Meanwhile, there exist dozens of commercial internet chat groups in which users—without much concern and worries—self-disclose about their mental health (e.g., depression, eating disorder, anxiety, etc.), about highly socially normed and often stereotyped habits (e.g., drug, drinking, dietary or eating habits, weight concerns, etc.) and even about suicidal tendencies. Studies focusing on data sharing behavior in social networks such as Facebook (www.facebook.com) show that the user’s belief or trust in the social media service is associated with an increased willingness to self-disclose and share personalized data (e.g., Dwyer et al., 2007). Thus, there is a high motivation of internet users to communicate and exchange personal and health-related data in social media platforms and online surveys. Trust of the user in the privacy protection of the service provider might promote this motivation.

Nevertheless, not every single user decides and behaves the same. There exists intra-individual and inter-individual variance in self-disclosure behavior. Evidence from a few studies so far indicate a positive relationship between certain personality traits such as impulsivity and risk taking, and self-disclosure behavior and the user’s sensitivity towards data privacy (Egelman and Peer, 2015), the latter being measured for example, by the degree of the user’s willingness to accept cookies from internet sites for the purpose of using services for self-disclosure (Coventry et al., 2016). The studies suggest that the user’s affective, cognitive, and motivational state as is expressed in the personality of the user personality constitute significant implicit driving forces for inter-individual differences in self-disclosure behavior and probably also in the degree to which self-disclosure behavior via the internet is sensitive to PA and TIP manipulations.

As it becomes obvious, human-centered privacy is a multifaceted concept (Acquisti and Grossklags, 2005; Hong and Thong, 2013; Smith et al., 2011) in which human factors and contextual factors dynamically interact. Still, there are open questions and a demand for research answering these questions scientifically under well-controlled experimental conditions. Specifically, how privacy awareness (PA) or trust in privacy (TIP), or a combination of both, PA and TIP, influence people’s motivation to self-disclose in specific contexts such as when sharing highly intimate, private data about their health and health behavior on the internet needs to be better understood. In the domain of health services, analog interventions that built upon face-to-face exchange of sensitive information and trust in confidentiality of the interaction partners (client/coach, patient/therapist) have moved towards digital solutions. Notably, these web- or internet-based health services are meanwhile frequently used by younger generations who are considered to be digital natives. In its worst case, unreflected self-disclosure of health information by this population group may have high negative impact and consequences in case of malicious data collectors. The group of younger adult internet users such as university students is particularly vulnerable because they are used to access online services for example for educational purposes or for taking part as volunteers in online research surveys. Moreover, as shown by previous and most recent studies conducted before and during the COVID-19 pandemic, university students might have a high motivation to seek for web-based health care and mental coaching programs on the internet to manage stress, depression, and anxiety symptoms, the latter having been observed of being of equal or even higher prevalence among university students compared to non-academic peer groups of similar age (e.g., Bewick et al., 2010; Davies et al., 2014; Stewart-Brown et al., 2000).

### 1.1 Aim of the Present Study

Building on the open questions outlined above, this study has the following major aims: first, investigate the relationship between self-disclosure behavior, privacy awareness (PA), and trust in privacy (TIP) among university students as digital natives and young adult internet users when taking part as volunteers in scientific online studies. Second, assess interindividual differences in self-disclosure behavior. Third, induce PA and TIP experimentally instead of using a purely self-report design (as in many previous studies). Fourth, investigate self-disclose behavior related to psychological domains of mental health, family, living, and academic live (for an overview see Table 1). Methodologically, from a psychological perspective, experimental designs as the one chosen in the present study might have restrictions in terms of ecological validity (for a discussion of validity in empirical software engineering see for example Freimut et al., 2002; Wohlin et al., 2012). However, as also agreed and suggested by other disciplines than Psychology (see Freimut et al., 2002; Wohlin et al., 2012), only an experimental design allows drawing causal inferences and going beyond correlational assumptions. Investigations among university students may lack generalizability to the general population. However, university students are an important part of the young adult population. As a population group, university students are often confronted with self-disclosure for science and education. In particular, taking part in studies as a volunteer is an important part in the curricula, e.g., in Psychology or Medicine. Moreover, as outlined above, university students might have a high interest in seeking for web-based health care. Therefore, the current investigation will give valuable insight into self-disclosure of private personal and health data, and aspects of data privacy among university students as a group of the population frequently sharing personal data on the internet via social media or by participating as volunteer in scientific studies.

**TABLE 1:** Questionnaires, scaling, and items with subscales and example items.

Questionnaire and scaling	Subscales and Example items

**Personality**	Personality traits:
Ten item personality inventory (TIPI; Gosling et al. (2003); German version by Muck et al. (2007))	- Extraversion
10 items	- Agreeableness
**Scaling:**	- Conscientiousness
1 (disagree strongly)	- Emotional Stability
2 (disagree moderately)	- Openness to Experiences
3 (disagree a little)	
4 (neither agree nor disagree)	
5 (agree a little)	
6 (agree moderately)	
7 (agree strongly)	
“No answer”	
**Privacy-I (Group A and D only)**	Concerns:
Privacy Concerns Scale (PCS; Buchanan et al. (2007)), first 18 items	- General caution
**Scaling:**	- Technical protection
1 (never)–5 (always)	
“Don’t know”	
“No answer”	
**Personal data (Sociodemographic)**	- Age: How old are you?
Single survey items	- Birthplace: What is your place of birth?
**Scaling:**	- Education: What is your job/course of study?
Open text	- Language: What is your first language?
“No answer”	- Residence: Where do you live?
	- Gender: What is your gender?
**Health (physical appearance and eating/drinking preferences)**	- Height: How tall are you (in cm)?
Single survey items	- Weight: What is your weight (in kg)?
**Scaling:** open text or “yes”/“no”	- Favorite food: What is your favorite food?
“Don’t know”	- Favorite drink: What is your favorite drink?
“No answer”	Health1: Do you exercise regularly?
	Health2: Do you drink alcohol regularly?
	Health3: Do you smoke?
**Self-concerns**	- Satisfaction with body: How satisfied are you with your own body?
Single survey items	- Satisfaction with oneself: How satisfied are you with yourself?
**Scaling:**	- Satisfaction with academia: How satisfied are you with your studies?
Percentage (0–100 in steps of 10)	- Satisfaction with your test performance: How satisfied are you with your test performance?
**Mental health**	Over the past 2 weeks, how often have you been bothered by any of the following problems?
Patient Health Questionnaire (PHQ-2; Löwe et al. (2005))	1. Little interest or pleasure in doing things
** Scaling:**	2. Feeling down, depressed, or hopeless
0 (not at all)	
1 (several days)	
2 (more than half the days)	
3 (nearly every day)	
“Don’t know”	
“No answer”	
**Privacy-II (Group A and Group D only)**	Concerns:
Privacy Concerns Scale (PCS; Buchanan et al. (2007)), items 19–23	- Technical protection
**Scaling:**	- General caution
1 (never)–5 (always)	
“Don’t know”	
“No answer”	
**Self-concept**	Please, describe yourself in terms of the following attributes, indicate to what degree the attribute is characteristic for you.
15 attributes-scale	
**Scaling:** 1–8	
“Don’t know”	
“No answer”	
** (memory control and attention check)**	Please describe yourself …
linguistic task: use 5 words to describe yourself (Herbert et al. (2021))	I am …
**Privacy-III (Group A and Group D only)**	Concerns:
Privacy Concerns Scale (PCS; Buchanan et al. (2007)), items 24–28	- Technical protection
**Scaling:**	- General caution
1 (never)—5 (always)	
“Don’t know”	
“No answer”	
**Social desirability**	Listed below are a number of statements concerning personal attitudes and traits. Read each item and decide how it pertains to you
Marlowe-Crowne Social Desirability Scale Crowne and Marlowe (1960), 7 items	
**Scaling:** 1–4 (instead of true/false)	
“Don’t know”	
“No answer”	
**Personality**	Please indicate how much the following statements apply to you
Revised Reinforcement Sensitivity Theory Questionnaire (rRST-Q; Reuter et al. (2016)), 31 items	
**Scaling:**	
1 (strongly disagree)	
2 (disagree)	
3 (agree)	
4 (strongly agree)	
“No answer”	
**Manipulation Check**	- How honest did you respond?
Self-disclosure	- Were the questions unpleasant for you?
**Scaling:** open text	- Would you have participated in this study if it was from a commercial provider on the internet?
Attention and memory check	- Would you trust a language assistant system (e.g., Alexa, Siri) with your responses to these questions?
**Scaling:** open text	- Do you remember the five attributes you noted down earlier in the survey to describe yourself? Please note them down once more
Self-Disclosure Motivation	- Did you participate in order to support the research project?
**Scaling:** „tick box and chose what holds true”	- Did you participate out of curiosity?
	- Did you participate out of boredom?
	- Did you participate out of sense of duty?
	- I don’t want to respond
	- Reason for participation: Other reason ___
**Context**	
** Scaling:**	On which device did you fill out the survey?
“Smartphone”	
“Tablet-PC”	
“Laptop or desktop-PC”	
“No answer”	
** Scaling:**	
Open text	In what context did you fill out the survey? Please write a comment about your decision concerning the choice of context
“I don’t want to answer”	

In summary, the following research questions (RQ) were investigated:

RQ1: To what extent are university students willing to self-disclose private and health data when taking part as volunteers in scientific online studies?
RQ2a: Do experimental manipulations of privacy awareness (PA) and trust in privacy (TIP) or a combination of PA and TIP promote or prevent self-disclosure behavior?
RQ2b: If so, which psychological domains and personal data (e.g., sociodemographic, living, academic life, health) will be most affected or probably differentially affected by the experimental manipulations?
RQ3: Is there a relationship in RQ1 and RQ2 with personality or gender or with interindividual differences?

## 2 Materials and Methods

### 2.1 Participants

**FIGURE 1:**
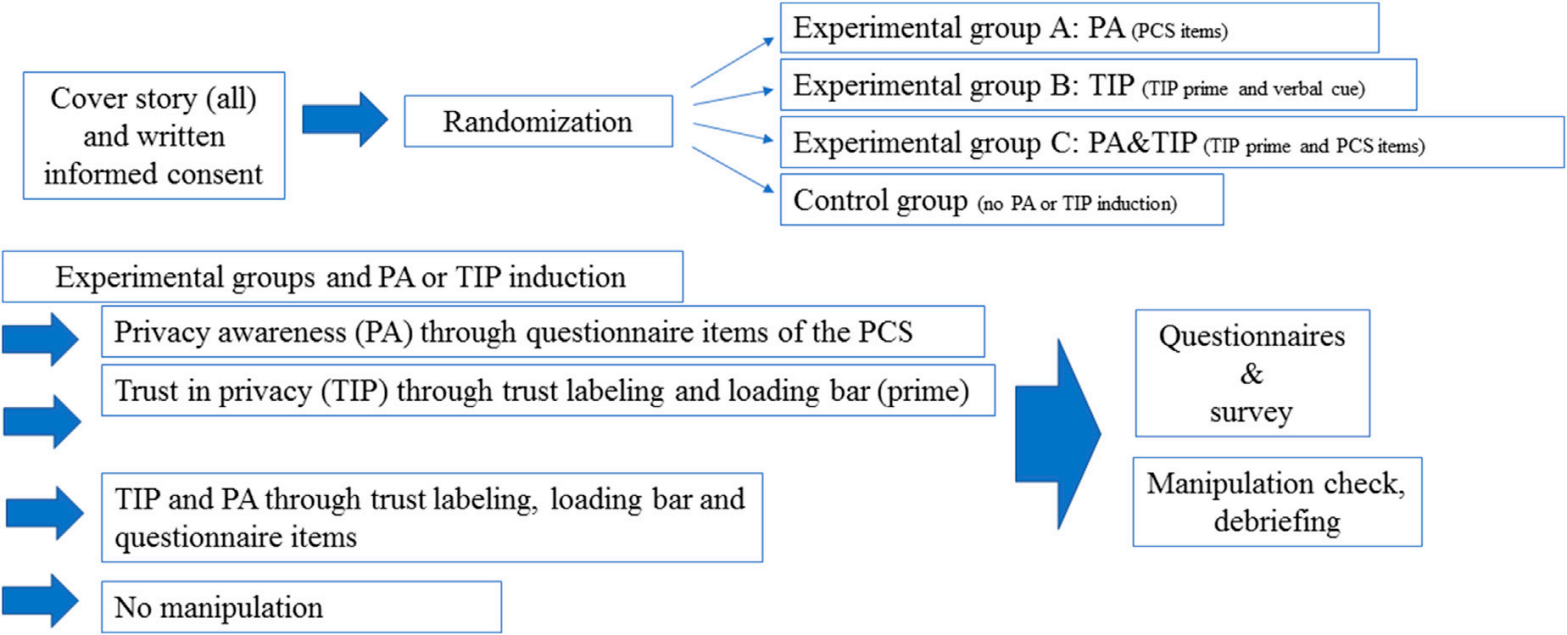
Overview of the study design and sequence of the online study. Note: PA, privacy awareness; TIP, trust in privacy; induction: participants were informed that data would be stored in a safe place and a loading bar was shown to simulate data storage in order to induce trust in privacy; cover story. For PA induction participants were shown items from the Privacy Concerns Scale (PCS; Buchanan et al., 2007) in order to induce privacy awareness.

The study was conducted at Ulm University, Germany. Data collection was hosted by the Department of Applied Emotion and Motivation Psychology and the Institute of Distributed Systems of Ulm University, Germany. The study and study design were approved by the local ethics committee (https://www.uni-ulm.de/einrichtungen/ethikkommission-der-universitaet-ulm/). The online survey was administered via LimeSurvey (https://www.limesurvey.org/de/). High proficiency in the German language as well as a minimum age of 18 years were inclusion criteria for study participation. The study was advertised via university mailing lists. Interested volunteers could indicate their study participation and register via the online platform SONA (https://uulm.sona-systems.com/Default.aspx?ReturnUrl=/), an online university management system for study and participant registration. In total, N = 546 volunteers registered for the study. Of these, *n* = 305 dropped out immediately after registration and did not provide informed consent, leaving a study sample of N = 241 volunteers who provided written informed consent prior to participation. 4.15% (*n* = 10) of these participants dropped out during the survey, 2.08% (*n* = 5) left after having completed the survey items of the Ten Item Personality Inventory (TIPI, Gosling et al., 2003; German version by Muck et al., 2007), 1.66% (*n* = 4) participants left after having completed the survey items “trait-other,” and 0.42% (*n* = 1) participants dropped out after having completed the Marlowe-Crowne Social Desirability Scale (Crowne and Marlowe, 1960). N = 231 participants completed the survey. Drop-out rates (immediate drop out and drop-out during the survey) did not differ significantly across the four experimental groups (PA, TIP, PA&TIP, control group, see Figure 1), *p* = 0.632 [Fisher’s exact test]. From N = 231 volunteers, *n* = 4 participants were excluded because they were no students. The final sample size for the analysis was N = 227 (34 men, 189 women, and *n* = 4 participants preferring giving “no answer” or “other” regarding items about gender). The mean age of the final study sample was: *M* = 21.95 years (*SD* = 3.91 years).

### 2.2 Procedure and Experimental Study Design of the Online Study

The assignment of volunteers to the four experimental conditions (PA, TIP, PA&TIP, control group, see Figure 1) was randomized. After drop-outs, a total of 50 university students (*n* = 7 men, *n* = 41 women, *n* = 2 no answer) took part in the experimental condition A (PA), *n* = 43 university students (*n* = 6 men, *n* = 37 women) took part in the experimental condition B (TIP), *n* = 79 university students (*n* = 12 men, *n* = 65 women, *n* = 1 non-binary, *n* = 1 no answer) took part in the experimental condition C (PA&TIP), and *n* = 55 university students (*n* = 9 men, *n* = 46 women) took part in the experimental condition D (control condition). For an overview of the study design, see Figure 1. Table 2 gives an overview of the participant sample and their assignment to the four experimental conditions. As shown in Table 2, there was no significant difference in the number of women and men taking part in the four experimental groups, χ2 (3) = 0.13, *p* = 0.988 [Pearson’s chi square test]. However, overall, more women finally completed the survey than men. There was no significant difference between experimental groups in mean age, *F* (3,218) = 0.05, *p* = 0.987 [one-way analysis of variance]. Also, the distribution of pursued academic degree (master, bachelor, etc.) did not differ significantly across the four experimental groups, *p* = 0.390 [Fisher’s exact test].

**TABLE 2:** Number and demographics of participants (women and men) in the final sample of the four experimental groups.

	Group			
	PA	TIP	PA&TIP	Control group
Sample size	*n =* 50	*n* = 43	*n* = 79	*n* = 55
Sex (*n*)	Male: *n* = 7	Male: *n* = 6	Male: *n* = 12	Male: *n* = 9
	Female: *n* = 41	Female: *n* = 37	Female: *n* = 65	Female: *n* = 46
	No answer: *n* = 2	No answer: *n* = 0	Non-binary: *n* = 1	No answer: *n* = 0
			No answer: *n* = 1	
Mean age (*SD*)	21.78 (4.48)	21.95 (2.91)	22.00 (4.36)	22.04 (3.38)
	No answer: *n* = 1	No answer: *n* = 1	No answer: *n* = 0	No answer: *n* = 3
Education (*n*)	Psychology: *n* = 46	Psychology: *n* = 39	Psychology: *n* = 70	Psychology: *n* = 49
	Computer science: *n* = 4	Computer science: *n* = 4	Computer science: *n* = 4	Computer science: *n* = 5
	Other courses of study: *n* = 0	Other courses of study: *n* = 0	Other courses of study: *n* = 5	Other courses of study: *n* = 1

PA, privacy awareness; TIP, trust in privacy. The number of “no answer” on each variable indicated how many participants preferred to not self-disclose this information.

**FIGURE 2:**
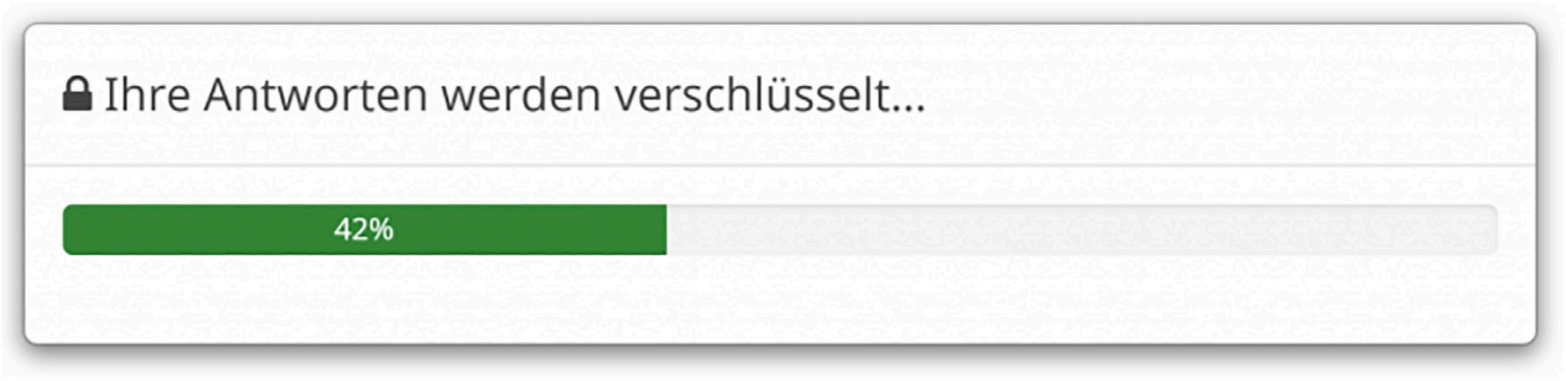
Data encryption box for visual TIP priming (experimental group B). English translation of the German statement: Your answers are being encrypted.

As shown in Figure 1, at the beginning of the survey, all participants received the same cover story, irrespective to which of the four experimental groups they were assigned. They were told that they are taking part in a scientific study conducted by two departments, one from the Institute of Psychology and Education of Ulm University and the other from Computer Science of Ulm University, Germany. They were informed that the aim of this joint scientific research project from Psychology and Computer Science is to develop smart virtual agents (such as Siri or Alexa), able to recognize and understand human feelings, that are empathetic with the human user’s health and thoughts, and infer the user’s intentions from the written and linguistic answers the users provide in online surveys. The participants were told, that for this purpose, the volunteers of this study will be asked to self-disclose information about very private, personal, and health-related aspects of their life. All volunteers were debriefed that there is no physical or mental risk (no pain or mood will be induced), that their data will be stored for scientific purpose only, that they can withdraw from the study any time without negative consequences, and that they take part in the study voluntarily. Participants who were randomly assigned to the experimental group A (privacy awareness, PA) next received questions from the Privacy Concerns Scale (PCS) (Buchanan et al., 2007). The PCS measures three facets of human-centered privacy, i.e., privacy-related attitudes (scale: Privacy Concern, e.g., question: “Are you concerned about who might access your medical records electronically”), privacy behaviors related to two scales (General Caution and Technical Protection), and asking for data protection behavior (e.g., “Do you clear your internet browser history regularly?”). Participants of group A received part of the items of the privacy concern subscale of the PCS as primes to trigger privacy awareness (PA) before filling in any of the questionnaires and survey items. In addition, in the experimental group A, items from the other two subscales of the PCS were inserted in the online survey as further priming questions to keep awareness high across the survey. In the experimental group B, trust in privacy (TIP) was experimentally induced by buzzwords in the debriefing instructions as well as by encryption prompts as visual TIP primes. Participants received detailed information about data storage. They were told that their data is stored on secure servers of the departments, and that data will be fully encrypted before its storage, i.e., converted to an encrypted code that can be decoded only by the scientific staff of the study. Any time the participants of group B (TIP) had filled in survey items or a questionnaire, a visual bar popped up on the computer screen (see Figure 2) to convince and remind the participants of this safe way of data encryption and storage of their data. Figure 1 illustrates an overview of the study design of the online survey. The participants of group C (PA&TIP) received both, the experimental privacy treatment of group A to induce PA as well as the experimental privacy treatment of group B to induce TIP. Participants of group D served as control group. This group (group D) received only the cover story and did not receive any PA or TIP treatment. This means that no PA or TIP was triggered or experimentally induced in the design of the group D before or while filling in the survey questions and questionnaires.

#### 2.2.1 Survey Items and Questionnaires

The survey items and questionnaires were the same for all participants irrespective of their assignment to one of the four experimental groups. All participants received the same closed and open-ended survey items and filled in the same psychological questionnaires asking about personal data, mental health, and or personality (see Table 1 for an overview). In addition, the Marlowe-Crowne Social Desirability Scale was included (MCSD, Crowne and Marlowe, 1960). Social desirability is a construct that measures a person’s tendency to answer in a socially acceptable way rather than reporting the “true” answer if the “true” answer would reflect the person’s attitudes, concerns, behaviors, personality, or emotions in a less socially favorable way. Psychological research identified topics particularly vulnerable to social desirability: personal income and earnings, self-attribution, feelings of low self-worth and/or powerlessness, religion, physical appearance in terms of size, shape or weight, consumption of drugs and alcohol, smoking, eating and drinking habits, socioeconomic status, biological and social sex, to name but a few examples that were therefore also included as health-related questions in the present survey. Table 1 provides a full summary and description of the survey items and questionnaires included in the survey. Of note, in Table 1 the survey items and questionnaires are clustered into psychological domains and listed in chronological order as they appeared in the online survey. At the end of the survey, all participants were fully debriefed about the experimental manipulation and about the purpose of the study (investigation of self-disclosure and data privacy behavior). Debriefing included self-reports about privacy concerns and privacy awareness. In addition, a manipulation check was included: participants were asked to answer questions about their motivation to participate in the survey by choosing among the following reasons: 1) to support science, 2) curiosity, 3) boredom, 4) sense of duty, 5) prefer not to say, or 6) other. They were also asked about the location (i.e., whether they filled in the survey e.g., at home on their private computers vs. at work). The participants were asked how open and honest they had answered the survey items, and also how open they would have answered the survey items if the survey would not have been for scientific purpose and not hosted by the university but provided by a commercial health provider. Finally, the participants were asked if they would have been willing to self-disclose if the survey had been entirely controlled by an artificial intelligence like Siri or Alexa instead of human scientists. The open items had to be answered by filling in a blank survey box. The participants could answer the questions or leave the survey box open (blank) in case they decided to prefer not to provide a response. The closed survey items (besides the items asking for physical appearance) and all questionnaire items had to be answered by clicking one of a number of possible answers, see Table 1 for an overview. As shown in Table 1, the number of possible answers included the response options “don’t know” or “no answer.” These two answering options allow to investigate the hypotheses related to RQ1–RQ3. Only, the items of the personality questionnaire measuring the Big Five personality dimensions included only one alternative answer option “no answer.” This is because the scales of the questionnaire already include an option of uncertainty (answer option: “neither applicable nor inapplicable”). Participants were informed that they will not be reimbursed individually but that they could take part in an online raffle and win a voucher. In addition, psychology students (undergraduates) could also choose to earn credit points for their bachelor degree. N = 154 psychology students were reimbursed with credit points.

#### 2.2.2 Hypotheses Sorted According to Research Questions

Hypothesis 1 (RQ1): It was hypothesized that university students will display high self-disclosure regardless of the psychological domains (see Table 1). This high self-disclosure behavior will manifest in the participants’ response behavior such that across survey items participants will display a low bias and tendency to choose the alternative response option “no answer.”

Hypothesis 2 (RQ2): The participants’ tendency to self-disclose will be significantly modulated by the experimental induction of PA, TIP, or PA&TIP. Particularly, if PA priming is successful in changing self-disclosure behavior, this should bias the answers of the participants in group A (PA) towards being less open in their answers compared to the other experimental groups or the control group. No psychological domain specificity of PA was expected, i.e., participants of the group A (PA) should generally more often answer the survey items with “no answer” compared to the control group or the TIP group. As far as trust in privacy (TIP) is concerned, participants of group B (receiving the trust in privacy priming) were expected to significantly less often choose “no answer” compared to, for instance, the experimental group A or the control group on the survey items asking for personality, height and weight, alcohol consumptions, smoking or regular exercising, because these items are related to socially desirable behavior. In addition, this group was expected to give less socially desirable answers on the Marlowe-Crowne Social Desirability Scale (Crowne and Marlowe, 1960). As far as the group of participants receiving the combined induction of PA&TIP is concerned, it was explored whether combining PA and TIP would promote the privacy paradox, or produce a conflict in one’s motivation of unreflected self-disclosure. If so, it was expected that particularly participants of group C (who receive PA&TIP treatment) compared to the other experimental groups will differ in their answers to the questions asking 1) to have answered the survey honestly, 2) to also take part if the study was for commercial instead of scientific reasons and 3) also be willing to self-disclose if the survey had been controlled by an artificial intelligence.

Hypothesis 3 (exploratory): Also, the impact and role of human factors in self-disclosure were explored. In particular, whether personality measures, gender, or interindividual differences could impact self-disclosure and sensitivity to PA or TIP induction.

### 2.3 Data Analysis and Statistics

The survey data was preprocessed manually, i.e., sum scores were calculated for the questionnaires and the scores of the questionnaires and single items were coded for parametric and non-parametric statistical testing. The respective and appropriate statistical tests chosen for testing the individual hypotheses and for answering the research questions are mentioned in the Results section in the text in parentheses. Statistical tests and analyses were conducted with RStudio (Version February 1, 5033, R Studio Team, 2019). Assumptions of parametric testing were examined. In case of violation of assumptions, non-parametric tests were chosen as alternative. Bonferroni correction was used in the event of multiple testing. *p*-values are reported at the significance level of *p* < 0.05.

## 3 Results

### 3.1 Self-Disclosure of Private and Health Data (Research Question 1/Hypothesis 1)

As far as overall self-disclosure of private and health data is concerned, it was expected that university students taking part voluntarily in the survey will show a low number of “no answer” or “don’t know” answers suggesting little concern and worry about data privacy among the participants. In support of this, participants showed in general a very little number of choices for “no answer” to the survey items asking for personal information (sex, age, place of birth, weight, height). The same was true for the survey items asking for individual preferences including food choices and drinking behavior (alcohol consumption). As shown in Table 3, the survey item asking for height received the most “no answer” answers (8.81%) followed by questions about favorite food (5.29%) and drink (5.29%). There was no shift towards reduced self-disclosure for the survey items asking for self-concerns such as satisfaction with one’s self, one’s physical appearance (in terms of body size and body shape), or one’s academic performance. The same holds true for the survey items asking for health-related behavior habits: only two out of 227 (0.88%) participants did not report their satisfaction with their own physical appearance or academic life. One participant (0.44%) did not report satisfaction with overall Self (i.e., Satisfaction with oneself, see Table 3). Five participants (2.20%) did prefer giving “no answer” for the item asking for satisfaction with the own academic performance and only one participant (0.44%) preferred the “no answer” for the items asking about alcohol consumption or regular exercise (physical activity). Amongst the items shown in Table 3, the survey items asking for alcohol consumption and exercising had the highest rate of “don’t know” answers. Two participants (0.88%) chose “no answer” for the item asking about smoking. The answer option “don’t know” was chosen by nine participants (3.97%) for exercising, eleven participants (4.85%) gave “don’t know” for alcohol consumption and three participants (1.32%) for smoking. The participants’ response tendencies did not change for those survey items asking for mental health. As summarized in Table 3, in total, only one participant (0.44%) answered the screening questions for depressive symptoms from the PHQ-2 questionnaire (Löwe et al., 2005) with the option “no answer.” “Don’t know” was chosen by one participant (0.44%) for the first depressive item asking for feelings of “little interest or fun” during the last 2 weeks, whereas two participants (0.88%) chose “don’t know” for the second depressive item asking about feelings of “hopelessness” during the last 2 weeks. Amongst the items shown in Table 3, the survey items asking for alcohol consumption and exercising had the highest rate of “don’t know” answers.

**TABLE 3:** Number of „no answer“ for the survey items asking for personal data for all participants (total) and across experimental groups.

Items	Group				
	PA *n* = 50	TIP *n* = 43	PA&TIP *n* = 79	Control *n* = 55	Total *n* = 227
Sex	2	0	1	0	3
Age	1	1	0	3	5
Place of birth	3	1	0	0	4
Native language	0	0	0	0	0
Residence	4	1	0	0	5
Height	5	9	0	6	20
Weight	5	3	1	1	10
Favorite food	3	3	4	2	12
Favorite drink	4	3	3	2	12
Satisfaction with body/physical appearance	1	0	0	1	2
Satisfaction with oneself	1	0	0	0	1
Satisfaction with studies (academic)	1	1	0	0	2
Satisfaction with test performance/academic life	2	1	1	1	5
Physical activity	0	1	0	0	1
Alcohol consumption	1	0	0	0	1
Smoking	0	0	0	2	2
PHQ-2 Item 1	0	1	0	0	1
PHQ-2 Item 2	0	1	0	0	1
TIPI Extraversion 1	0	0	0	1	1
TIPI Extraversion 2	0	0	0	0	0
TIPI Agreeableness 1	0	0	0	0	0
TIPI Agreeableness 2	1	0	2	3	6
TIPI Conscientiousness 1	0	1	0	2	3
TIPI Conscientiousness 2	0	0	0	0	0
TIPI Emotional Stability 1	0	0	0	0	0
TIPI Emotional Stability 2	1	0	0	1	2
TIPI Openness to Experiences 1	0	0	1	1	2
TIPI Openness to Experiences 2	0	0	0	0	0
Total	35	27	13	26	101

PA, privacy awareness; TIP, trust in privacy. *Patient Health Questionnaire-2* (PHQ-2; Löwe et al., 2005), *Ten Item Personality Inventory* (TIPI; Gosling et al., 2003; German version by Muck et al., 2007).

### 3.2 Self-Disclosure of Private and Personal Health-Related Information Under Experimental Induction of Privacy Awareness And/Or Trust in Privacy vs. Control (Research Question 2/Hypothesis 2)

It was hypothesized that experimental induction of PA and/or TIP will influence the tendency to self-disclose private and health data. In total, the participants of the PA group had the highest number of “no answers,” followed by the TIP group and the control group. The PA&TIP group had the lowest total number of “no answer” choices (see Table 3). Moreover, as shown in Table 4, the privacy awareness group (PA group) showed the highest number of “no answer” (*M* = 0.54, *SD* = 1.66) in comparison to all other experimental groups, (TIP group: *M* = 0.49, *SD* = 1.20; PA&TIP group: *M* = 0.11, *SD* = 0.51) including the control group (*M* = 0.26, *SD* = 0.62) for the survey items asking for personal information including sex, age, and place of birth (see items in the section “Personal data” in Table 1). Statistical comparisons between the experimental groups showed a significant difference between experimental groups in the number of “no answer” answers for the items containing personal data, *H* (3) = 8.58, *p* < 0.05 [Kruskal-Wallis test; for statistics the number of “no answer” answers was summarized across the items]. The post hoc analysis showed, that the TIP group (*M* = 0.49, *SD* = 1.20) and the PA&TIP group (*M* = 0.11, *SD* = 0.51) differed significantly from each other, *p* < 0.05.

**TABLE 4:** Means and standard deviations of number of “no answer” and “don’t know” in dependence of experimental group as well as corresponding analysis of variance (ANOVA) or Kruskal-Wallis test.

		Group				ANOVA/Kruskal-wallis test	
		PA n = 50	TIP *n* = 43	TIP + PA *n* = 79	Control *n* = 55	*F* (df1, df2), *p*-value/*H* (df), *p*-value
Personal data	No answer	0.54 (1.66)	0.49 (1.20)	0.11 (0.51)	0.26 (0.62)	*H* (3) = 8.58, *p* < 0.05
Self-concerns	No answer	0.10 (0.58)	0.05 (0.31)	0.01 (0.11)	0.04 (0.19)	*F* (3,223) = 0.76, *p* = 0.519
Health questions	No answer	0.02 (0.14)	0.02 (0.15)	0.00 (0.00)	0.04 (0.19)	*F* (3,223) = 0.87, *p* = 0.457
	Don’t know	0.06 (0.24)	0.07 (0.26)	0.10 (0.30)	0.16 (0.42)	*F* (3,223) = 1.14, *p* = 0.335
Patient health questionnaire-2	No answer	0.04 (0.28)	0.00 (0.00)	0.00 (0.00)	0.00 (0.00)	*F* (3,223) = 1.18, *p* = 0.317
	Don’t know	0.00 (0.00)	0.02 (0.15)	0.01 (0.11)	0.02 (0.14)	*F* (3,223) = 0.37, *p* = 0.779
Ten-item personality inventory	No answer	0.04 (0.20)	0.02 (0.15)	0.04 (0.25)	0.15 (0.56)	*F* (3,223) = 1.56, *p* = 0.199
Trait-self	No answer	0.00 (0.00)	0.05 (0.31)	0.00 (0.00)	0.00 (0.00)	*F* (3,223) = 1.44, *p* = 0.234
	Don’t know	0.04 (0.28)	0.02 (0.15)	0.01 (0.11)	0.04 (0.19)	*F* (3,223) = 0.29, *p* = 0.836
Trait-other	No answer	0.00 (0.00)	0.05 (0.31)	0.19 (1.69)	0.00 (0.00)	*F* (3,223) = 0.55, *p* = 0.650
	Don’t know	0.08 (0.45)	0.56 (2.64)	0.06 (0.40)	0.16 (0.74)	*F* (3,223) = 1.67, *p* = 0.175
Marlowe-Crowne social desirability scale	No answer	0.06 (0.43)	0.05 (0.33)	0.00 (0.00)	0.00 (0.00)	*F* (3,206) = 0.95, *p* = 0.415
	Don’t know	0.08 (0.35)	0.03 (0.16)	0.01 (0.12)	0.04 (0.20)	*F* (3,206) = 1.06, *p* = 0.367
BIS/BAS revised reinforcement sensitivity theory questionnaire	No answer	0.50 (1.23)	0.65 (1.23)	0.51 (1.25)	0.49 (1.33)	*F* (3,223) = 0.17, *p* = 0.918
Total	No answer	1.35 (2.52)	1.32 (2.83)	0.82 (2.14)	0.88 (1.44)	*H* (3) = 3.41, *p* = 0.332
	Don’t know	0.27 (0.71)	0.73 (2.84)	0.18 (0.48)	0.43 (1.03)	*H* (3) = 3.06, *p* = 0.382

PA, privacy awareness; TIP, trust in privacy. *Ten-Item Personality Inventory* (Gosling et al., 2003; German version by Muck et al., 2007), *Marlowe-Crowne Social Desirability Scale* (Crowne and Marlowe, 1960), *BIS/BAS, Revised Reinforcement Sensitivity Theory Questionnaire* (Reuter et al., 2016), health questions concerning exercising, alcohol consumption and smoking, *Patient Health Questionnaire-2* (Löwe et al., 2005).

For the survey items asking for self-concerns (see section “Self-Concerns” in Table 1 for an overview), the PA group (*M* = 0.10, *SD* = 0.58) showed the highest number of “no answer” answers, followed by the TIP group (*M* = 0.05, *SD* = 0.31), and the control group (*M* = 0.04, *SD* = 0.19). The PA&TIP group (*M* = 0.01, *SD* = 0.11) had the lowest mean number of “no answer” on self-concerns/satisfaction. However, these differences were not significant between groups, *F* (3,223) = 0.76, *p* = 0.519 [one-way analysis of variance]. Similarly, there were no significant group differences for the items asking for health (see section “Health” in Table 1 for an overview): descriptively, the control group had the highest number of “no answer” and “don’t know” answers, the PA&TIP group showed the lowest number of “no answer” choices and the PA group for “don’t know” (see Table 4), but differences were not significant (for an overview see Table 4). Analysis of the screening questions for depressive symptoms (PHQ-2) did not yield any significant differences in “no answer” choices between the four experimental groups, *F* (3,223) = 1.18, *p* = 0.317 [one-way analysis of variance], and also not in “don’t know” answers between groups, *F* (3,223) = 0.37, *p* = 0.779 [one-way analysis of variance]. The number of “no answer” and “don’t know” answers was low in all four experimental groups (see Tables 3, 4).

For the personality items of the TIPI questionnaire, the control group had the highest number of “no answer” answers (*M* = 0.15, *SD* = 0.56), followed by the PA group (*M* = 0.04, *SD* = 0.20) and the PA&TIP group (*M* = 0.04, *SD* = 0.25), and the TIP group (*M* = 0.02, *SD* = 0.15). Again this difference was not significant between the four experimental groups, *F* (3,223) = 1.56, *p* = 0.199 [one-way analysis of variance]. Table 4 also summarizes the number of “no answer” and “don’t know” answers for the items belonging to the Marlowe-Crowne Social Desirability Scale (Crowne and Marlowe, 1960), the BIS/BAS Revised Reinforcement Sensitivity Questionnaire (Reuter et al., 2016), and all other survey items asking for personality. Statistical comparisons yielded no significant differences between groups, all *p* > 0.05. For all questionnaires listed in Table 4, the highest number of “no answer” was given by the PA group and the TIP group. The combined experimental group (PA&TIP) had the lowest number of “no answer” choices. However, these differences were not significant between groups, *H* (3) = 3.41, *p* = 0.332 [Kruskal-Wallis test]. With regard to “don’t know” answers, the TIP group had the highest number, while the combined experimental group (PA&TIP) had the lowest number. No significant difference emerged, *H* (3) = 3.06, *p* = 0.382 [Kruskal-Wallis test].

### 3.3 Impact of Personality and Gender on Self-Disclosure of Personal and Health Information (Research Question 3/Hypothesis 3)

There were no significant effects of personality or gender on the participants’ self-disclosure of personal and health information, and on all items together for preferences of the response options “no answer” and “don’t know” (see Table 4). However, as described above in Section 3.2, the total number of “no answers” differed significantly between the TIP group and the PA&TIP group on disclosure of personal information. Therefore, “personality” and “gender” were further explored in the experimental group receiving the trust in privacy induction (TIP group) or the trust in privacy and privacy awareness induction (PA&TIP group) as possible human factors influencing self-disclosure after experimental induction of TIP or PA&TIP, respectively. The interaction effect between the personality trait “openness” (as assessed by the TIPI) (Gosling et al., 2003; German version by Muck et al., 2007) and the group variable (TIP or PA&TIP) was significant, CI95 [0.06; 0.18] [Bootstrapping according to Field et al., 2012, pp. 298–301]. The TIP group showed less self-disclosure of personal information with higher openness scores and the PA&TIP group showed higher self-disclosure of personal information.

The interaction effect of the personality trait BAS and group on disclosure of personal information was also significant, CI95 [−0.56; −0.09] [Bootstrapping according to Field et al., 2012, pp. 298–301]. There was a higher number of self-disclosures for personal information with increasing BAS scores in both groups (TIP and PA&TIP), and especially for the PA&TIP group. The interaction effects of gender and the other personality traits were not significant, gender: CI95 [−0.83; 0.26] [Bootstrapping according to Field et al., 2012, pp. 298–301]; TIPI extraversion: CI95 [−0.28; 0.19]; TIPI emotional stability: CI95 [−0.21; 0.36]; TIPI conscientiousness: CI95 [−0.46; 0.25]; TIPI agreeableness: CI95 [−0.29; 0.23]); BIS: CI95 [−0.12; 0.99]; FFFS: CI95 [−0.22; 2.15] (TIPI: Gosling et al., 2003; German version by Muck et al., 2007; BIS/BAS, Revised Reinforcement Sensitivity Theory Questionnaire: Reuter et al., 2016). For an overview see Table 5.

**TABLE 5:** Regression of personality and gender on disclosure of personal and health information and general disclosure over all items.

		Disclosure		
Questionnaire	Subscale	Personal and health information “no response”	Answer option “no answer”	Answer option “don’t know”
Ten-Item Personality Inventory	Extraversion	*H* (12) = 9.32, *p* = 0.675	*H* (12) = 5.79, *p* = 0.926	*F* (1,200) = 1.52, *p* = 0.220
	Agreeableness	*H* (8) = 5.25, *p* = 0.731	*H* (8) = 8.05, *p* = 0.428	*H* (8) = 9.63, *p* = 0.292
	Conscientiousness	*H* (11) = 9.43, *p* = 0.582	*H* (11) = 12.04, *p* = 0.361	*F* (1,200) *=* 0.93, *p =* 0.337
	Emotional stability	*H* (12) = 10.21, *p* = 0.598	*H* (12) = 13.12, *p* = 0.360	*F* (1,200) = 0.51, *p =* 0.476
	Openness to experience	*H* (8) = 4.98, *p* = 0.760	*H* (8) = 8.00, *p* = 0.433	*H* (8) = 3.30, *p* = 0.914
BIS/BAS revised reinforcement sensitivity theory questionnaire	BIS	*H* (27) = 24.09, *p* = 0.625	*H* (27) = 25.50, *p* = 0.546	*F* (1,159) = 1.00, *p =* 0.320
	BAS	*H* (18) = 24.46, *p* = 0.141	*F* (1,159) = 0.04, *p = 0.850*	*H* (17) = 16.33, *p* = 0.501
	FFFS	*H* (24) = 19.15, *p* = 0.744	*F* (1,159) = 0.41, *p = 0.522*	*H* (23) = 28.69, *p* = 0.191
Sex		*H* (1) = 0.96, *p* = 0.326	*H* (1) = 1.25, *p* = 0.264	*F* (1,204) = 1.10, *p = 0.297*

BIS, behavioral inhibition system; BAS, behavioral activation system; FFFS, fight flight freezing system. Ten-Item Personality Inventory (Gosling et al., 2003; German version by Muck et al., 2007), BIS/BAS, Revised Reinforcement Sensitivity *Theory Questionnaire* (Reuter et al., 2016). “No response” refers to the option to leave *open text format (answer format) blank.*

### 3.4 Further Exploration (Research Question 3)

It was further explored whether interindividual differences could influence self-disclosure among university students.

#### 3.4.1 Depression

Self-reported depressive symptoms as screened with the PHQ-2 (Löwe et al., 2005) did not significantly differ between the four experimental groups, *H* (3) = 4.44, *p* = 0.218 [Kruskal-Wallis test]. Descriptively, mean PHQ-2 scores were higher for the PA&TIP group (*M* = 2.23, *SD* = 1.60) and the control group (*M* = 2.22, *SD* = 1.49) than for the PA group (*M* = 2.06, *SD* = 1.31) or the TIP group (*M* = 1.86, *SD* = 1.79). When split according to cut-off scores (PHQ-2 sum score higher or lower than 2) the participants below the cut-off score showed a higher number of “no answer” answers (*M* = 1.18, *SD* = 2.86, *Mdn* = 0.00) and a lower number of “don’t know” answers (*M* = 0.27, *SD* = 0.57, *Mdn* = 0.00) compared to the participants above the PHQ-2 cut-off score (“no answer”: *M* = 0.58, *SD* = 1,37, *Mdn* = 0.00, “don’t know”: *M* = 0.37, *SD* = 1.47, *Mdn* = 0.00). There was, however, no significant difference between groups, in “no answer”: *W* = 3,324, *p* = 0.112, or “don’t know”: *W* = 3,040, *p* = 0.575 [Wilcoxon rank-sum test used]. On average, the mean PHQ-2 score was below the clinical cut-off score of the PHQ-2 (cut-off of 3) in all four experimental groups, suggesting a healthy study sample.

#### 3.4.2 Social Desirability and Privacy Concern (Manipulation Check)

Differences in social desirability were also explored. The participants of the four experimental groups did not differ significantly in self-reported social desirability as assessed with the Marlowe-Crowne questionnaire (Crowne and Marlowe, 1960), *F* (3, 197) = 1.61, *p* = 0.188 [one-way analysis of variance]. Descriptively, the PA group (*M* = 17.73, *SD* = 3.25), the PA&TIP group (*M* = 17.74, *SD* = 3.88) and the participants in the TIP group (*M* = 16.14, *SD* = 4.08) or the control group (*M* = 17.16, *SD* = 3.94) had almost comparable mean scores.

Statistical analysis of the privacy concern items of the manipulation check (see manipulation check in Table 1) that were provided to all participants, yielded no significant difference between the four experimental groups, *H* (3) = 4.10, *p* = 0.251 [Kruskal-Wallis test] (PA: *M* = 3.64, *SD* = 0.94; TIP: *M* = 3.98, *SD* = 1.01; PA&TIP: *M* = 3.86, *SD* = 1.04; Control: *M* = 3.91, *SD* = 0.06).

#### 3.4.3 Reasons for Study Participation

Analysis of the items about the motivation of study participation showed that 58.59% (*n* = 133) of the participants reported to have taken part in the study to support the research project. 35.24% (*n* = 80) replied to have taken part for reasons of curiosity, and 8.37% (*n* = 19) replied to have taken part for other reasons such as boredom. A total of 11.01% (*n* = 25) reported to have felt a sense of duty concerning participation. Only 3.97% (*n* = 9) did not want to give a response to this question and 50.66% (*n* = 115) of the participants stated other reasons for participation. Across all reasons, most of the participants (*n* = 100) replied to have taken part because of receiving credit points for their bachelor degree. However, overall, participants of the four experimental groups did not differ in their reasons for study participation, “support of research project”: *χ* ^*2*^ (3) = 1.45, *p* = 0.694 [Pearson’s chi square test], “curiosity”: *χ* ^*2*^ (3) = 0.56, *p* = 0.905 [Pearson’s chi square test], “boredom”: *p* = 151 [Fisher’s exact test because of expected frequency below 5], “sense of duty”: *p* = 0.954 [Fisher’s exact test], “no answer”: *p* = 0.735 [Fisher’s exact test], “other reasons”: *χ* ^*2*^ (3) = 0.67, *p* = 0.881 [Pearson’s chi square test]. Items asking participants to describe themselves in own words were used as memory check to determine participants’ accuracy and processing depth of the survey.

## 4 Discussion

This study investigated the willingness of university students to self-disclose via the internet private and health-related information when taking part as volunteers in online scientific studies. In association with this, it was explored whether self-disclosure behavior can be changed by experimental induction of privacy awareness (PA), trust in privacy (TIP), or a combination of both (PA&TIP). Of note, as described in Section 2, privacy awareness (PA) and trust in privacy (TIP) were induced implicitly by visual priming and by TIP or PA sensitive instructions. There was no explicit mention to the participants that these primes, items, and information were meant to trigger or induce PA or TIP or both to not bias the participants’ response behavior. Participants in each of the experimental groups including the control group received detailed debriefing about the privacy purpose of study only after the completion of the survey. However, ethically, written informed consent requires providing volunteers with information about the purpose of the survey (non-commercial but scientific), about voluntariness and anonymity of study participation, the right to withdraw any time without undue reservation, and, last but not least, about the basic principles of data security and protection. In sum, this information which was given to all participants at the beginning of the survey might have already triggered privacy awareness (PA) and trust in privacy (TIP) in all participants irrespective of the further experimental manipulations of PA, TIP, or PA&TIP. In line with this speculation, the participants of all four experimental groups showed high self-disclosure behavior, irrespective of which experimental group they were assigned to. This high self-disclosure behavior occurred although participants had the chance to refrain from disclosure by choosing for nearly each survey item additional answer options such as “no answer” or “don’t know.” Previous studies suggest that offers of “no answer” as response option lead to a shift in the participants’ response behavior towards reduced self-disclosure even without any additional PA or TIP priming (for an overview Joinson and Pane, 2007). In the present study, this tendency towards “no answer” or “don’t know” answers as indicators of reduced willingness of self-disclosure was not systematically observed. In total, only *n* = 84 of the N = 227 participants (37.00%) who completed the survey did make use of the answer option “no answer” and *n* = 52 (22.91%) made use of the option “don’t know” at least once or up to 15 times (“no answer”: 12.40%, “don’t know”: 30.61%) from a total number of 121 survey items offering these two options for “no answer” and 49 items offering these options for “don’t know.”

Of note, the overall drop-out rate of those participants withdrawing from the survey showed that 305 participants dropped-out immediately after registration and ten participants dropped-out during the course of the study corresponding to drop-out rates predicted from the literature for online studies with university students as volunteers (Hoerger, 2010). According to this literature, about 10% of subjects drop out within 100 survey items, and an additional 20% drop out after 500 survey items (Hoeger, 2010). With respect to drop outs, motivation for study participation was higher than expected from the literature concerning drop-out rates, and drop-out rates did not differ between the four study groups (PA, TIP, PA&TIP, or control). Therefore, it is unlikely that the experimental manipulation influenced reasons for dropping-out.

The motivation to self-disclose and to finish study participation can only partly be explained by monetary incentive because study participation was not reimbursed financially or individually. Nevertheless, participants could win vouchers or, for psychology students only, get study credit points. 154 students from the 204 (75.49%) psychology students taking part in the survey were undergraduates asking for credit points. Indeed, when asked for the reason to participate (see manipulation check in Table 1 and Results in Section 2), the offer of receiving credit points turned out to be one of the major reasons for taking part in the study for undergraduate students from Psychology. This suggests that receiving academic incentives for study participation in terms of credit points can affect the motivation of study participation. Analysis of “no answer” choices did, however, not differ as a function of pursued academic degree, *H* (2) = 5.52, *p* = 0.063; albeit a trend could be detected. Analysis of the “don’t know” answers did again not differ as a function of the academic degree of the students, *H* (2) = 4.38, *p* = 0.112.

Theoretically, self-disclosure behavior has been explained by psychological theories of planned behavior (Dienlin and Trepte, 2015; Trepte et al., 2020). In this theoretical view, the participants’ final decision of whether to disclose personal information or not is dependent on motivational and affective-cognitive factors such as the participants’ beliefs about the outcome of their behavior, the normative value of the behavior, and the possibility of self-control. Asking participants about their motivation to participate in the study showed that the majority (58.59%) out of the N = 227 participants selected 1) “to support the research project”, 2) 35.24% for reasons of “scientific curiosity,” 3) 11.01% of the participants replied to have felt a “sense of duty,” and (as mentioned above) 49.02% of the undergraduate students from Psychology replied to have been motivated by receiving credit points. In addition, on a scale from zero to 100% conviction, only 12.34% of the study participants replied that they would have taken part if this survey was provided by a commercial provider (answers 50 and higher) and only 15.86% replied that they would have taken part if this survey was controlled by an artificial intelligence (answers 50 and higher). Statistical comparisons of the distribution of answers across the different motivational reasons for study participation were, however, not significant between the four experimental groups. Accordingly, outcome and normative beliefs (supporting science and getting reward for it while being a “good student” (sense of duty)) seem to be major motivational driving forces among university students for showing high self-disclosure behavior. These motivational factors seem so strong across the psychology domains (personal data, health-related data, or personality data) that the final decision to answer honestly instead of choosing an alternative response (e.g., “no answer”) cannot be changed by any of the privacy manipulations: whether PA, TIP, or the combination of both (PA&TIP), neither of the three experimental manipulations was systematically associated with a significantly stronger tendency of the participants in the PA, TIP, or PA&TIP groups to refrain from self-disclosure as compared to the control group receiving no PA, TIP, or PA&TIP induction. Statistical analysis of “no answer” answers showed only a few significant differences between the experimental groups, even if items were split into psychological domains. One of the few significant differences between experimental groups was that the TIP group and the PA&TIP group differed significantly from each other in the number of “no answer” choices for personal data: the TIP group showed more often “no answer” choices than the PA&TIP group.

Although previous studies reported a gender bias in self-disclosure behavior and a tendency towards higher willingness to disclose personal and health-related information in women than in men (e.g., Dindia and Allen, 1992; Bond, 2009), gender effects could not be confirmed in the present study when looking at “no answer” or “don’t know” answer options (see Results). Likewise, the personality of the participants turned out to have no significant impact on self-disclosure choices of “no answer” answers across the experimental groups, but “openness” as a personality trait seemed to play a role when participants were primed with trust in privacy (TIP group) or trust in privacy and privacy awareness (PA&TIP group). Also, BAS (sensitivity to reward) as a personality trait seemed to influence self-disclosure when participants were primed with trust in privacy (TIP group) or with both, trust in privacy and privacy awareness (PA&TIP group). Although further studies are needed to investigate these effects, previous studies also suggested that certain personality traits such as impulsivity or risk taking may modulate self-disclosure behavior and the user’s sensitivity towards data privacy (Egelman and Peer, 2015).

Given the almost negligible impact of experimental PA, TIP, or PA&TIP induction on the participants’ self-disclosure behavior, one may ask whether social desirability might play a role or whether social desirable answers differed as a function of the induction of PA, TIP or PA&TIP. Analysis of mean scores of social desirability as assessed with the Marlowe-Crowne Social Desirability Scale (MCDS; Crowne and Marlowe, 1960) showed no significant differences between the participants of the four groups receiving experimental induction of either privacy awareness (PA), trust in privacy (TIP), or a combination of privacy awareness (PA) and trust in privacy (TIP) compared to the control group. The participants receiving PA, TIP, or PA&TIP induction did not show a significant increase or decrease in their choice of “no answer” answers compared to the control group. Notably, the two response options “no answer” or “don’t know” did not significantly differ between the participants of the four experimental groups. Thus, inducing PA by letting participants fill out privacy concern questions or inducing TIP by priming, or inducing both TIP and PA seemed not to bias the participants towards giving more or less favorable and socially desirable answers to protect or promote self-disclosure. In line with this, the participants’ self-reported answers to the question of how honest they had answered each of the survey items revealed (on a scale from 0–100) more than 90% of conviction in 98.68% of the participants.

Previous research on self-disclosure behavior on the internet partly provided support for a privacy paradox (Bedrick et al., 1998; Barnes, 2006). According to this paradox, users are willing to share private data for example on social media platforms despite privacy concerns. More recent research suggests that this paradox of unreflected self-disclosure behavior despite significant concerns about privacy needs to be specified. In particular, it has been suggested that the privacy paradox should not be considered independent from contextual and human factors that influence the user’s motivation and the user’s intentions of why, when, and where to self-disclose. Previous research identified the user’s privacy awareness (PA) or trust in privacy (TIP) to play an important role and theoretical models and meta-analytic studies (Jeff Smith et al., 2011) suggest a relationship between the user’s privacy concerns, privacy awareness and willingness to adopt privacy-protective behaviors. The results of the current study using implicit strategies of experimental PA or TIP manipulation does not support the hypothesis that making participants trust in privacy or increasing their attention towards privacy awareness affects the users’ self-disclosure behavior in the context of their participation as volunteers in scientific studies. Although the present study might have limitations (see below), the results of little or no impact of PA, TIP, or PA&TIP on university students self-disclosure behavior should be taken seriously. As outlined and discussed above, the results suggest that university students as a population group are vulnerable to privacy disclosures and probably also to the privacy paradox, especially when taking part as a volunteer in a research study. Future studies could follow-up on this observation and investigate the present study design and self-disclosure behavior outside the university context.

## 5 Limitations and Future Outlook

The generalizability of the results of this experimental online study might have limitations. Psychologically, limitations in generalizability can be classified according to challenges to validity. This classification is also well known and accepted in other disciplines (e.g., see Freimut et al., 2002; Wohlin et al., 2012 for a discussion of threats to validity in empirical software engineering research). As far as generalizability is concerned, first, one may ask whether the results are representative for all university students, including women and men, and all students pursuing different academic careers. Although significance testing did not reveal any gender effects nor any effect of pursued academic career (being a student of Psychology or Computer Science), there were more women than men, and more undergraduate and graduate students from Psychology than e.g., from Computer Science participating in the study (see Table 2). Nevertheless, the distribution of women and men in the present study sample reflects the typical distribution of percentage of women and men among psychology students and most of the participants in the present study were psychology students. Still, it would be interesting in future research to balance study participation across pursued academic career and gender. Second, the observed high tendency of self-disclosure behavior among the full participant sample (irrespective of group assignment) might produce ceiling effects making it difficult to detect subtle differences between the experimental groups. Nevertheless, the high willingness to self-disclose makes university students a vulnerable target group for whom to develop strategies for privacy control. Luckily, self-disclosure behavior and trust in privacy seem to be highly context dependent, i.e., the minority of study participants replied to have taken part if the survey was from commercial providers or controlled by artificial intelligence. Of course, this should be explored further given that health services usually including face-to-face contact between therapists or coaches and clients, are already supported by digital e-health solutions and already frequently used by the younger generation. To this end, it would be worth investigating if the current findings are representative for university students who are consulting for health services. Analysis of e.g., depression screenings in the present study sample showed a mean score below clinical scores. Although exploratory, when groups were split according to cut-off scores, those self-reporting depressive symptoms above the cut off scores showed a lower (though not statistically significant) tendency to use “no answer” as option of choice.

## Data Availability

The raw data supporting the conclusion of this article will be made available by the authors, without undue reservation.
